# Mono-institutional phase 2 study of innovative Stereotactic Body RadioTherapy targeting PArtial Tumor HYpoxic (SBRT-PATHY) clonogenic cells in unresectable bulky non-small cell lung cancer: profound non-targeted effects by sparing peri-tumoral immune microenvironment

**DOI:** 10.1186/s13014-019-1410-1

**Published:** 2019-11-26

**Authors:** Slavisa Tubin, Mohammad K. Khan, Gerardo Salerno, Waleed F. Mourad, Weisi Yan, Branislav Jeremic

**Affiliations:** 1 KABEG Klinikum Klagenfurt, Institute of Radiation Oncology, Feschnigstraße 11, 9020 Klagenfurt am Wörthersee, Austria; 2 Department of Radiation Oncology, Emory University School of Medicine, Winship Cancer Institute, 1365-C Clifton Road, 30322 Atlanta, NE Georgia; 3 Department of Neurosciences, Mental Health and Sensory Organs / Department of Clinical and Molecular Medicine, Universita’ La Sapienza Roma, Ospedale Sant’ Andrea, Via di Grottarossa, 1035, 00189 Rome, RM Italy; 4 Markey Cancer Center, Department of Radiation Medicine, University of Kentucky Lexington ky, UK Medical Center MN 150, Lexington, KY 40536-0298 USA; 5 Department of Radiation Oncology, Thomas Jefferson University, 111 S 11th St, Philadelphia, PA 19107 USA; 6 BioIRC, R&D Center for Biomedical Research, Kragujevac, SERBIA and Research Institute of Clinical Medicine, 13 Tevdore Mgvdeli St, 0112 Tbilisi, Georgia

**Keywords:** Novel unconventional SBRT, Partial irradiation, Bystander effect, Abscopal effect, Tumor hypoxia, Immune microenvironment

## Abstract

**Background:**

Radiotherapy-induced lymphopenia may be limiting the success of therapy and could also negatively affect the ability of immune system in mediating the bystander (BE) and abscopal effects (AE). A novel SBRT-based PArtial Tumor irradiation of HYpoxic clonogenic cells (SBRT-PATHY) for induction of the tumoricidal BE and AE by sparing the peritumoral immune microenvironment and regional circulating lymphocytes has been developed to enhance the radiotherapy therapeutic ratio of advanced lung cancer. The aim of this retrospective review of prospectively collected mono-institutional phase 2 study was to compare the outcomes between unconventional SBRT-PATHY and standard of care in unresectable stage IIIB/IV bulky NSCLC.

**Materials and methods:**

Sixty patients considered inoperable or unsuitable for radical radio-chemotherapy were enrolled and treated using the following 3 regimens: SBRT-PATHY (group I, *n* = 20 patients), recommended standard of care chemotherapy (group II, *n* = 20 patients), and institutional conventional palliative radiotherapy (group III, *n* = 20 patients).

**Results:**

Median follow-up was 13 months. The 1-year overall survival was 75, 60, and 20% in groups 1, 2 and 3, respectively (*p* = 0.099). The 1-year cancer specific survival was 90, 60, and 20% in groups 1, 2, and 3, respectively (*p* = 0.049). Bulky tumor control rate was 95% for SBRT-PATHY compared with 20% in the other two groups. BE and AE were seen by SBRT-PATHY in 95 and 45% of patients, respectively. Multi-variate analysis for cancer specific survival was significant for treatment effect with SBRT-PATHY (*p* < 0.001) independent of age, sex, performance status, histology, stage, treated bulky site and tumor diameter. SBRT-PATHY resulted in lower toxicity (*p* = 0.026), and improved symptom control (*p* = 0.018) when compared to other two treatment options.

**Conclusion:**

SBRT-PATHY improved treatment outcomes in unresectable NSCLC and should be investigated in larger trials.

Present study has been retrospectively registered on 8th of August 2019 by the ethic committee for Austrian region „Kärnten “in Klagenfurt (AUT), under study number A 31/19.

## Highlights

- SBRT-PATHY is a novel approach for induction of the bystander and abscopal effects
- This study is retrospective review of prospective mono-institutional phase 2 trial
- SBRT-PATHY vs. standard of care in population with T4-unresectable bulky NSCLC
- Bystander and abscopal effects induced by SBRT-PATHY were 95% and 45%, respectively
- SBRT-PATHY improved survival and tumor control compared to standard of care.

## Introduction

The management of advanced NSCLC is challenging due to various patient- and tumor related factors [1–6], with tumor size and volume being among the more important ones [7–10]. This is especially true in T4-unresectable bulky NSCLC where standard concurrent radio-chemotherapy (RT-CHT) may neither improve survival nor offer an improvement in quality of life [11–13]. Even with modern RT planning, it is not possible to deliver adequate ablative radiation dose in a safe way using conventional approaches. Better local control and survival is shown in early stage NSCLC when SBRT was utilized. However, the application of total tumor volume SBRT in bulky NSCLC is limited by surrounding tissue tolerance.

The real value of the immunomodulatory effects of RT have yet to be defined. The potential beneficial immune stimulating properties of RT have been questioned by many scientists in the past and present, with a renewed focus on the immune-mediated abscopal effects (AE) [14] as well as on the depletion of lymphocytes [15, 16]. Recent studies have described an association between the RT-induced lymphopenia with poor oncologic outcome, with the thought that RT using larger volumes/multiple daily fractions can lead to global immunosuppression [17–20]. RT-induced lymphopenia may be limiting the success of therapy in many tumor types [21–24] and could also negatively affect the ability of immune systems in mediating the RT-induced non-targeted effects (NTE). NTE of RT are rare, unintentional and exclusively observed as immune-mediated clinical phenomena that could have great therapeutic potential. There are two types of NTEs: 1) abscopal (systemic) effect of local radiation observable as regression of distant unirradiated tumor site, and 2) the bystander (local) effect (BE), which happens when irradiation of only one tumor part induces regression of the surrounding tumor tissue that was not targeted with radiation. The first clinical evidences of AE were described in the 1950s [25], while the first experimental evidences of BE emerged in the 1990s [26]. The underlying mechanisms behind NTE remain speculative. So far, “immune-mediated”, “cytokine-based tumor-signaling” and “pseudo-abscopal” mechanistic theories have been proposed [27].

Here we describe the first clinical series that provides evidence of AE and BE in bulky NSCLC patients with unfavorable characteristics treated with a non-conventional concept of **SBRT**-based **PA**rtial **T**umor irradiation targeting exclusively **HY**poxic clonogenic cells (**SBRT-PATHY**). The preclinical and clinical findings of this translational cancer research showed for the first time that the hypoxic tumor segment in respect to normoxic tumor cells, if selectively irradiated as inductor of NTE, express higher potential for the induction of NTE and increased tumor control, while minimizing side effects [28–31]. In addition, SBRT-PATHY focuses on the sparing of peritumoral immune microenvironment plus regional circulating lymphocytes in efforts to enhance BE and AE for improved locoregional tumor control [32]. Our concept implies that for immune modulation, the entire tumor volume may not need to be radiated: only a partial tumor, to initiate the immune cycle in radiation-spared peritumoral immune environment could be enough for bulky NSCLC.

The aim of this retrospective review of prospectively collected mono-institutional phase 2 study was to compare the treatment outcomes in terms of survival, tumor control and toxicity of SBRT-PATHY vs. traditionally used palliative treatments in population affected by unresectable bulky NSCLC.

## Methods and materials

We conducted Institutional Review Board approved study to evaluate if SBRT-PATHY can improve outcomes in bulky NSCLC patients over palliative CHT and conventional RT. Eligibility criteria included adult patients with T4-unresectable bulky NSCLC (≥6 cm) deemed unsuitable for radical RT-CHT due to its volume and site. Table 1 shows patients and treatment characteristics. 60 patients were enrolled and treated per protocol using the following 3 regimens:

**Table 1:** Patient and disease characteristics

Characteristic:	No. (%)			*p*-value
SEX:	SBRT-PATHY	CHT	PALLIATIVE RT	
Male	11 (55)	17 (85)	11 (55)	0.070
Female	9 (45)	3 (15)	9 (45)	
AGE(years):				
Median/range	68.7/41–88	66.9/51–86	74.2/56–88	0.080
ECOG PERFORMANCE STATUS:				
0–1	12 (60)	15 (75)	8 (40)	0.078
2–3	8 (40)	5 (25)	12 (60)	
HISTOLOGY:				
Adenocarcinoma	8 (40)	12 (60)	8 (40)	0.340
Squamous	12 (60)	8 (40)	8 (40)	0.340
Other (mixed or no histology)	0 (0)	0 (0)	4 (20)	***0.013***
NSCLC STAGE:				
IIIB	12 (60)	7 (35)	11 (55)	0.247
IV	8 (40)	13 (65)	9 (45)	
TREATED BULKY SITE:				
Central	14 (70)	15 (75)	11 (55)	0.377
Peripheral	6 (30)	5 (25)	9 (45)	0.380
LLr	1 (5)	2 (10)	2 (10)	0.804
LLl	6 (30)	2 (10)	5 (25)	0.279
ULr	8 (40)	11 (55)	9 (45)	0.626
ULl	5 (25)	5 (25)	4 (20)	0.911
UNRESCTABLE BULKY NSCLC:				
diameter mean/range (cm)	8.1/6–17	7.4/6–12	8.0/6–13	***0.047***
BULKY-RELATED SYMPTOMS:				
Dyspnea	15 (75)	11 (55)	14 (70)	0.377
Cough	9 (45)	9 (45)	10 (50)	0.935
Pain	12 (60)	6 (30)	10 (50)	0.153
Haemoptysis	2 (10)	2 (10)	6 (30)	0.147
Two or more symptoms	12 (60)	10 (50)	14 (70)	0.435

Abbreviations: ECOG-Eastern Cooperative Oncology Group, NSCLC-non small cell lung cancer, SBRT-PATHY-SBRT PArtial Tumor irradiation of the HYpoxic segment,LLr-lower lobe right, LLl-lower lobe left, ULr-upper lobe right, ULl-upper lobe left, BTV-bystander tumor volume (hypoxic segment), CHT-chemotherapy, N.A.- not applicable

GROUP 1: SBRT-PATHY exclusively to the bulky tumor (20 patients),

GROUP 2: recommended standard of care CHT (20 patients),

GROUP 3: conventional palliative RT of 30 Gy in 10 fractions exclusively to the bulky tumor (20 patients).

The patients were not randomized. The decision relative to the treatment group was done in the institutional lung tumor board based on the age, comorbidities, performance status or patient personal choice.

### Target definition for SBRT-PATHY

**Fig. 1:**
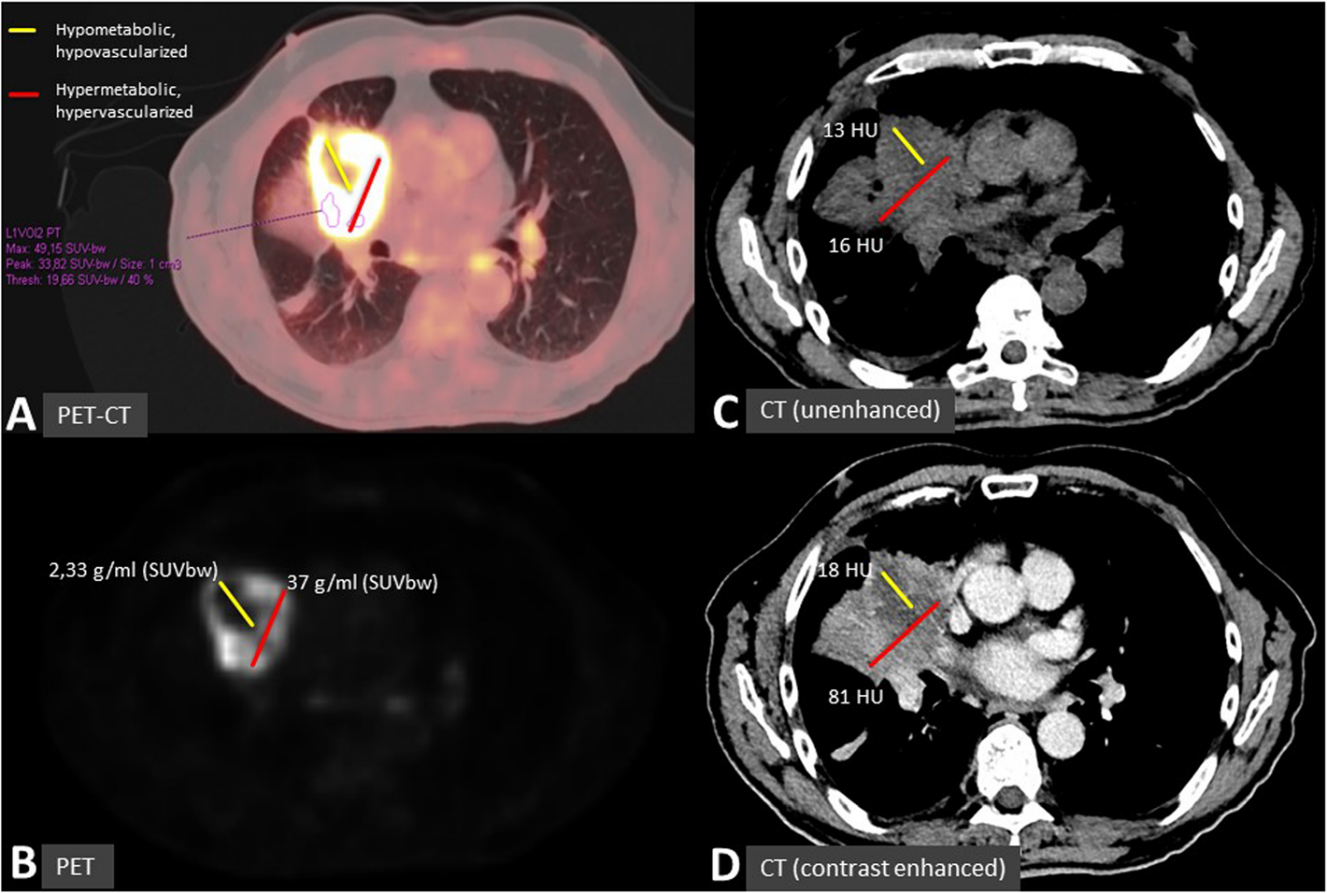
**a-d** Definition of target for SBRT-PATHY: BTV, or “hypoxic“ tumor segment refers to the combined 18F-FDG PET-CT (**a**, **b**) and contrast-enhanced CT (**d**) image findings, derived from the SUV and HU values. This to capture the junctional, contrast-hypo-enhanced (i.e. no significant increase in HU-values after contrast medium injection: **c** vs. **d**) and hypometabolic (SUV-value of ≤3 and/or ≤ 30% of SUVmax) tumor regions between the central-necrotic and the remaining peripheral-vascularized and hypermetabolic tumor segments

**Fig. 2:**
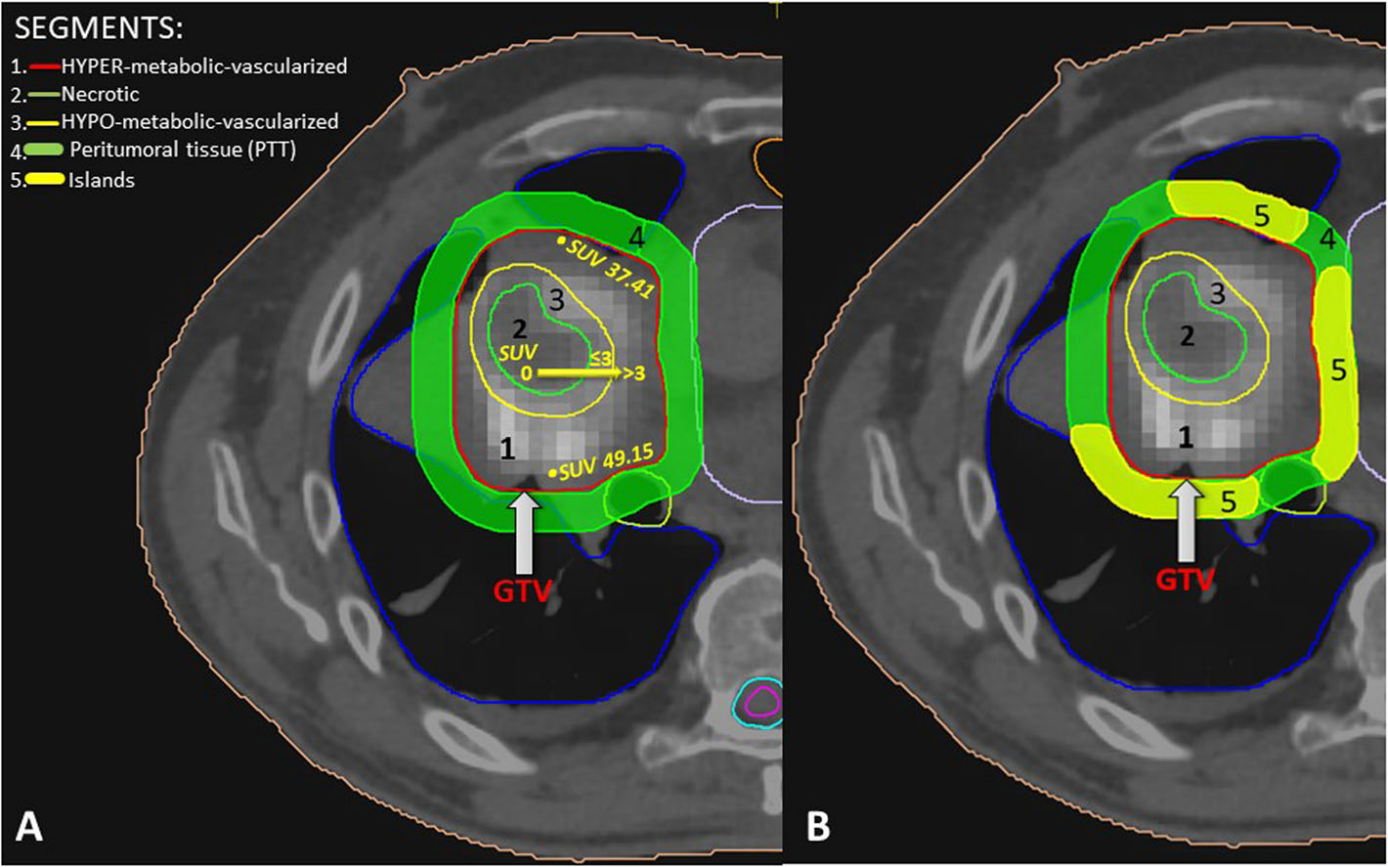
**a, b** Target definition by segmentation of an unresectable bulky lung cancer (**a**): Segment 1- representing the contrast-enhanced (vascularized, “normoxic”) peripheral tumor segment, (GTV), Segment 2 - being the contrast-unenhanced (necrotic, “anoxic”) central tumor region, Segment 3- delineating the contrast-hypo-enhanced (hypovascularized, “hypoxic”) junctional tumor region as an up to a maximum of 5 mm junctional zone between the central-necrotic and the remaining peripheral-vascularized tumor segments, Segment 4 - representing peritumoral tissue; (**b**) Sparing of the peri-tumoral-surrounding immune microenvironment (thick green line) by creating as an organ at risk multiple “immune microenvironmental islands” (thick yellow line) in order to spare from radiation dose a significant volume (for ex. 2/3) of the tumor microenvironment, leaving it intact and functional

**Fig. 3:**
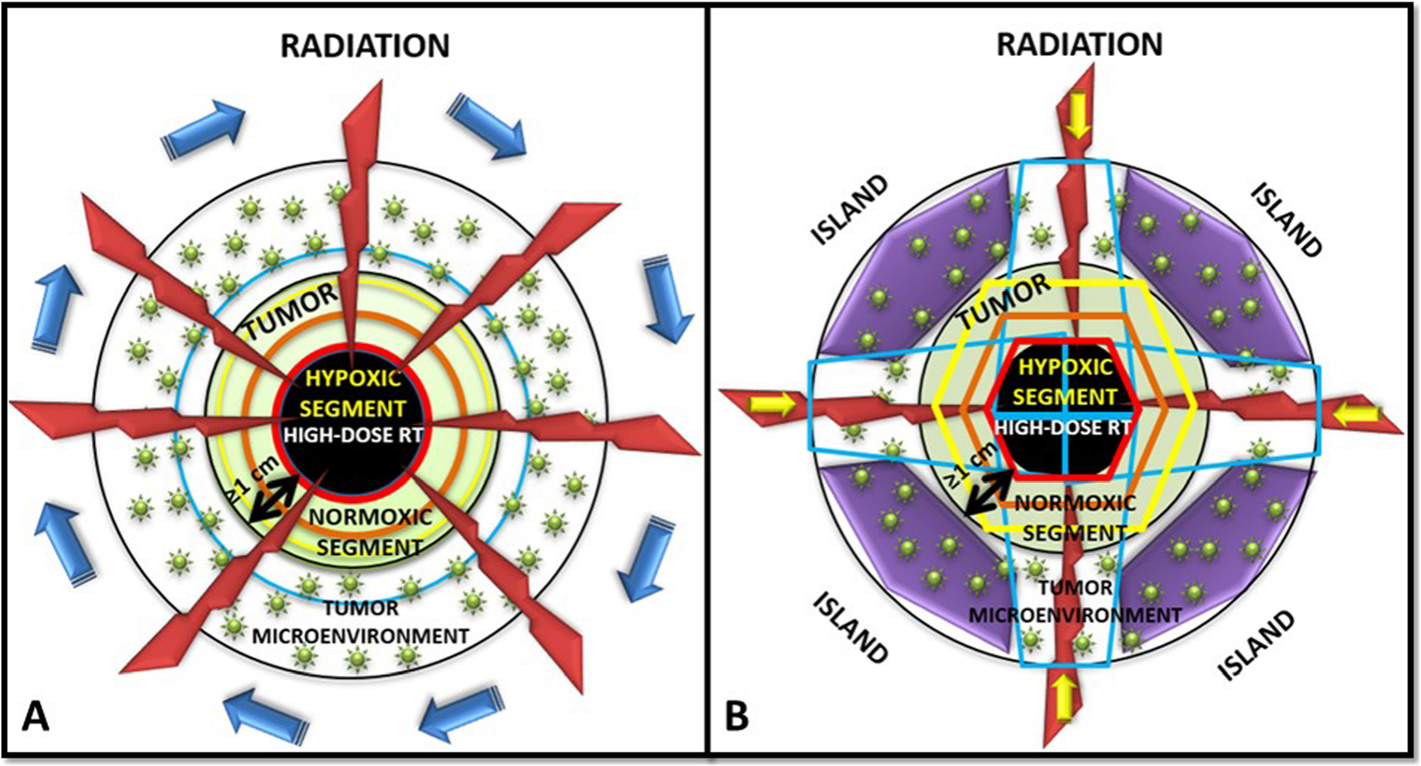
**a, b** Techniques for precise and feasible radiation dose delivery to the “hypoxic“ tumor segment while sparing the peri-tumoral-surrounding immune microenvironment: figure shows how a desired high radiation dose (red ring) is delivered by the mean of dynamic arcs (**a**) or multiple static radiation fields (**b**) very precisely and conformally to the hypoxic tumor segment (black area). Moving from that high-dose region through the normoxic tumor towards outside of the tumor, the dose will sharply fall off (orange ring-moderate dose, yellow ring-low dose) reaching an acceptably low dose-level (blue ring in **a**, or volume delineated by the blue lines in **b**) outside the tumor at the level of the tumor microenvironment. In this case, an acceptably low radiation dose will cover a small volume of the peri-tumor microenvironment closer to the tumor’s periphery leaving it intact and functional

Due to the lack of access to hypoxia-specific PET tracer, we used a combination of CT and 18F-FDG-PET to define the target for SBRT-PATHY. Patients underwent a 4D contrast enhanced simulation-CT using 2 mm slice thickness. Images were then transferred to Monaco-Elekta treatment planning system, and fused with PET-CT. Each bulky tumor was then divided into three segments (Fig. 1a-d, Fig. 2a):

- Segment 1- represented the contrast-enhanced (vascularized, “normoxic”) peripheral tumor segment,
- Segment 2*-* represented the contrast-unenhanced (necrotic, “anoxic”) central tumor region, and
- Segment 3- represented the contrast-hypo-enhanced (hypovascularized, “hypoxic”) tumor region as an up to a maximum of 5 mm zone between the central-necrotic and the remaining peripheral-vascularized tumor segments.
  PET was used to define the hypometabolic tumor volume (segment 3) between the necrotic and the peripheral hypermetabolic tumor segment. In order to define the boundaries of segment 3, we used Agfa HealthCare, IMPAX EE R20 XVII SU4 CD Viewers and Siemens Healthineers, Syngo.via system workstations to determine the tumor’s SUV 3-, or 30% of SUVmax- value volume that matched the previously defined “hypovascularized data” of CT (Fig. 1a-d, Fig. 2a). Our institutional experience suggested a steep SUV increase when moving from the necrotic tumor segment towards the peripheral hypervascularized tumor starting at SUV-value of approximately 3 and/or 30% of SUVmax. Thus defined segment 3 was then subtracted from the peripheral remaining hypermetabolic-vascularized (“normoxic”) tumor segment 1 to create the Bystander Tumor Volume (BTV) containing a hypovascularized and hypometabolic tumor volume (SUV ≤ 3, or ≤ 30% of SUVmax). The average GTV-BTV ratio was approximately 3:1: the prescribed radiation dose was delivered to the BTV which represented the 30% of the GTV (bulky tumor). No additional margins (neither Clinical Target Volume-CTV nor Planning Target Volume-PTV) were applied to the BTV. Once the BTV was created, the SBRT-PATHY planning also included sparing of the peri-tumoral-surrounding immune microenvironment. For that purpose, the peritumoral tissue (PTT), with the immune system cells as the mediators of the NTE of RT, has been considered as a new organ at risk (Fig. 2a, b, Fig. 3a, b). Considering the sharp fall off dose by using VMAT-SBRT techniques, for the purpose of maintaining the PTT-local immune system cells functionality, constraining of the dose at that level has been achieved by adding a uniform 1 cm margin to the GTV and then subtracting the same GTV to create the PTT (Fig. 2a, segment 4). The goal of the treatment planning was to keep the dose within the PTT as low as reasonably achievable, but considering the following dose constraints as primary objective: Dmin<1Gy/fraction, Dmax<5Gy/fraction, Dmean<3Gy/fraction. For those specific cases characterized by unfavorably irregular BTV form and/or its relationship with GTV, where the primary constraints can’t be met, the PTT was split into multiple “immune microenvironmental islands” in order to spare from radiation dose a significant volume (for ex. 2/3) of the tumor microenvironment leaving it intact and functional (Fig. 2b, Fig. 3b, segment 5). In this case, the localization of the “islands” has been chosen following the regional blood-lympho-vascular anatomy in order to create the island in proximity of the blood-lymph vessels and loco-regional lymph node stations (e.g. hilar and mediastinal area). The same previously mentioned PTT constraints were then applied to those islands.
  The PET-proven/suspicious metastatic lymph nodes and distant metastases if present, were intentionally not irradiated.
  Before each treatment, CBCT (XVI system, VERSA HD) was obtained to verify the isocenter. Considering that the hypoxic tumor segment cannot be identified on the CBCT images, for the purpose of SBRT-PATHY treatments, IGRT has been performed by matching the GTV between the simulation CT and the CBCT.

### Radiotherapy technique

Patients were immobilized using the BodyFix (Elekta AB, Stockholm, Sweden) and the breathing was limited with the use of BodyFix abdominal pressure. The patients were trained to breathe more regularly with the lowest achievable breathing amplitude to reduce the target movement and presence of artefacts in the 4DCT scan. SBRT plans were calculated using Monte Carlo algorithm. Dose prescription depended on tumor site and volume and was delivered to the BTV with VMAT by VERSA HD FFF (Elekta AB, Stockholm, Sweden) in 1–3 fractions each of 10–12 Gy to the 70% isodose-line (Dmax 14.5Gy per fraction). Among the patients treated with the single-fraction regimen, if after the first restaging at 1 month the remaining bulky volume was still large enough in order to define BTV, an additional adaptive single 10 Gy-fraction was delivered. In that case, treatment planning and delivery have been performed as previously described. With regard to the dose constraints, those reported in TG101 were used [33].

### Follow-up

Based on RECIST criteria, response to treatment was defined as a 30% or greater regression of bulky tumor. The first assessment of the radiological response was performed at 1 month after the treatment by using CT and/ or PET-CT, followed by repeated scans at month 2 and then every 3 months. Being the NTE exclusively radiation-related phenomena, BE was measured as the whole bulky tumor regression outside the targeted hypoxic segment exclusively after SBRT-PATHY treatment because of a partial bulky tumor irradiation. AE was applicable exclusively to the local treatments like SBRT-PATHY or conventional palliative RT as an eventual regression of the distant (metastatic) unirradiated tumor sites. Toxicity was evaluated using the CTCAE Criteria [34]. All procedures performed in the present study were in accordance with the ethical standards. All the patients signed the informed consent. Present study has been registered by the local ethic committee under study number A 31/19.

### Statistical analysis

The results of each group (1 = SBRT-PATHY, 2 = CHT, 3 = CONVENTIONAL PALLIATIVE RT) were correlated with clinic variables using tumor control as endpoint by Fisher’s exact test. The survival curves were generated by Kaplan–Meier method and their difference tested by the log–rank test. Multivariate analysis was performed to test independent influence of various variables. The R cran statistical software package (R version 3.5.3) -- “Great Truth “Copyright (C) 2019 The R Foundation for Statistical Computing) was employed for all analysis. The criterion for significance level was considered at *P* < 0.05.

## Results

*Patient and treatment characteristics* are given in Table 1. The three treatment groups were comparable in terms of performance status, histology and disease stage except that SBRT-PATHY group had tumors which were significantly larger than those in the other two groups (*p* = 0.047). The mean BTV volume was 65.3 mL (range 6.4–229.3 mL) corresponding to 34.2% of mean bulky volume of 190.7 mL (range 26.5–451.2 mL). For SBRT-PATHY, mostly, the prescribed dose to the BTV was 10–12 Gy × 1 to 70% (average Dmax 14.5 Gy) in 14 (70%) patients. In 9 of them (45% of total), an additional adaptive single 10Gy-fraction was delivered after the first restaging at 1 month in order to maximize the tumor control. Remaining 6 patients (30%) were treated with 10Gy × 3 to 70% (average Dmax 44.5Gy). 6/20 (30%) patients from SBRT-PATHY group were treated with CHT/immunotherapy before SBRT-PATHY, and all experienced disease progression. In the CHT group, Cis/Carboplatin in combination with Vinorelbine/Pemetrexed was mostly used, followed by Carboplatin/Gemcitabine/Necitumumab and Pembrolizumab-monotherapy. 8/20 (40%) patients were treated with second-line CHT while 4/20 (20%) also with third-line. As a second- or third-line CHT mostly Nivolumab, Taxotere and Pembrolizumab were prescribed. On an average, 4 cycles were in given in almost all patients (16/20, 80%) as first line CHT.

**Fig. 4:**
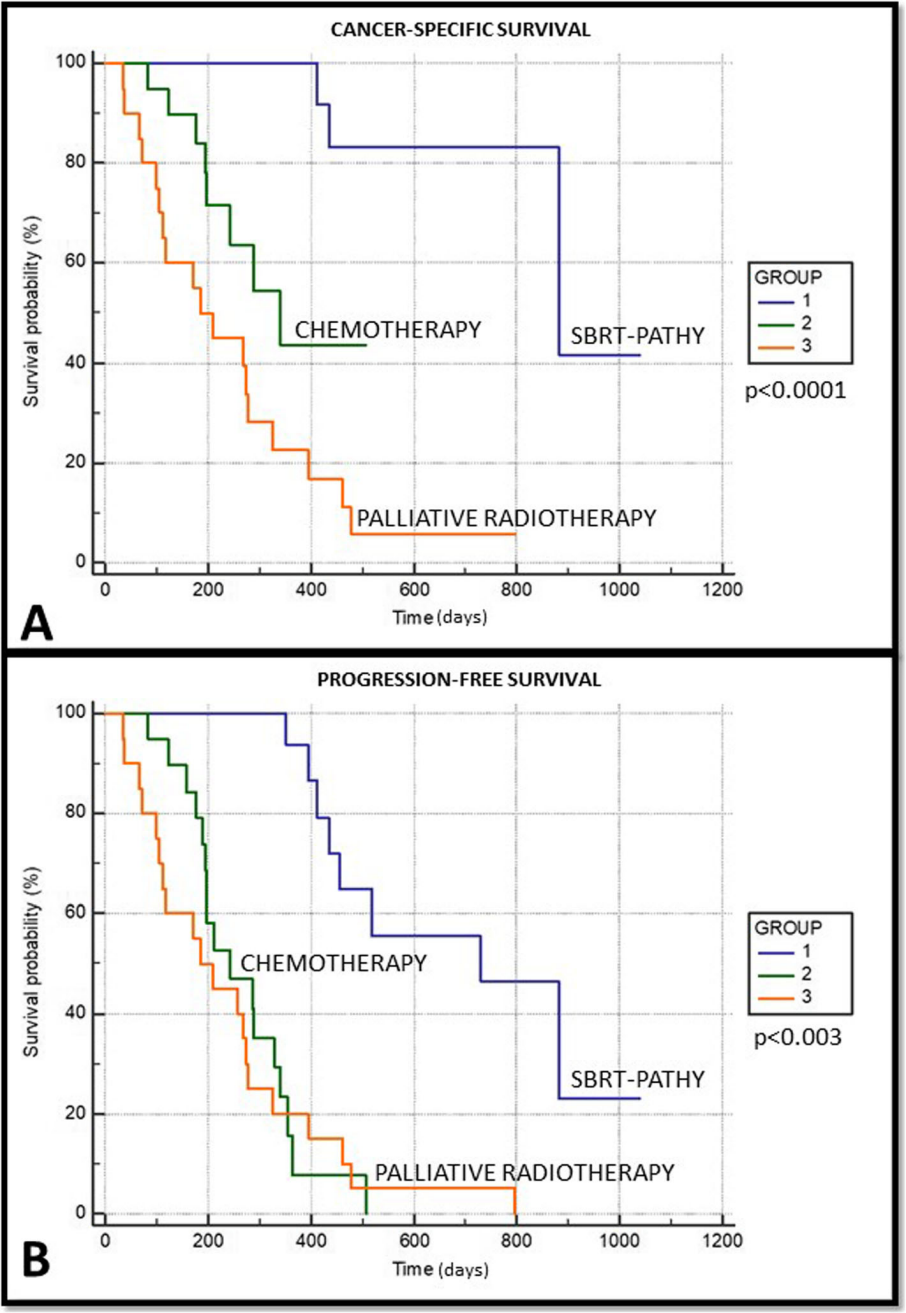
**a, b** Actuarial Kaplan-Meier cancer-specific (**a**) and progression-free survival (**b**) of 60 patients treated with three different regimens: gruop I-SBRT-PATHY, group II-CHT and group III-CONVENTIONAL PALLIATIVE RADIOTHERAPY

Tables 2 and 3 and Fig. 4, respectively summarize the main *clinical results*. The median follow-up time was 13 months (range 4–27 months). Bulky disease was more likely to respond to SBRT-PATHY being the bulky response rate 95% (19/20) for SBRT-PATHY while in other two groups it has been equally achieved in 20% of patients (*p* = 0.005). The median bulky tumor shrinkage was 68.9% (range 30–100%) with five (25%) complete responses after SBRT-PATHY but 40% for CHT and 47.5% for conventional palliative RT. Distant tumor response (measured as AE) was achieved exclusively with SBRT-PATHY in 45% (9/20) of patients. AE were observed at distant, unirradiated tumor sites in the lung, mediastinal and retroperitoneal lymph nodes with the median tumor shrinkage of 50% (range 30–100%). The probability of AE occurrence was related to intensity of BE being more probable for patients whose bulky tumors reduced more than 50% (*p* > 0.05). SBRT-PATHY was more likely to improve the survival in respect to CHT or palliative RT. The 1-year overall survival rates were 75% (median 353 days), 60% (median 217 days) and 20% (median 145 days) (*p* = 0.099), cancer-specific survival rates at 1 year 90, 60 and 20% (*p* = 0.049) and progression-free survival rates at 1 year 60, 15 and 0% (*p* = 0.003) for SBRT-PATHY, CHT, and palliative RT, respectively. The bulky-related symptoms represented by chest pain, dyspnea, coughing and hemoptysis were controlled in 80% of patients treated with SBRT-PATHY, and in 15 and 25% of those treated with CHT and palliative RT, respectively. Obviously, those symptoms were improved and better controlled with SBRT-PATHY than with other treatments (*p* = 0.018). Analysis of treatment-related toxicity showed that 15% of SBRT-PATHY patients developed grade 1 fatigue as the only observed toxicity, while 65% of those treated with CHT experienced more significant (grade 2–3) side effects like nausea and vomiting, diarrhea, pancytopenia, neuropathy and pneumonitis. In group III 15% of patients developed mild fatigue and dysphagia (grade 1). 45% of group II patients developed grade 2–3 leukopenia while no patients among other treatment groups experienced same side effect. As summarized, toxicities were significantly more frequent in CHT-treated patients, except dysphagia and pneumonitis which were not significantly different between the treatment groups, an important finding showing that SBRT-PATHY was virtually harmless (*p* = 0.026).

**Table 2:** Clinical results: comparative summary of the clinical results relative to the treatment group

CLINICAL OUTCOME	SBRT-PATHY (20 patients)	CHT (20 patients)	PALLIATIVE RT (20 patients)	p-value
BULKY RESPONSE (CR OR PR)	95% (19/20)	20% (4/20)	20% (4/20)	***0.005***
DISTANT TUMOR CONTROL	45% (9/20) (AE)	55% (11/20) (CHT)	0% (0/20) (AE)	***0.010***
OVERALL SURVIVAL at 1 year	75% (15/20)	60% (12/20)	20% (4/20)	0.099
CANCER SPECIFIC SURVIVAL at 1 year	90% (18/20)	60% (12/20)	20% (4/20)	***0.049***
PROGRESSION FREE SURVIVAL at 1 year	60% (12/20)	15% (3/20)	0% (0/20)	***0.003***
TOXICITY:	15% (3/20)	65% (13/20)	15% (3/20)	***0.026***
Fatigue	3 (15) Grade 1	13 (65) Grade 1–3	3 (15) Grade 1	***0.005***
Dysphagia	0 (0)	0 (0)	2 (10) Grade 1	0.126
Nausea/Vomiting	0 (0)	5 (25) Grade 2	0 (0)	***0.004***
Diarrhea	0 (0)	4 (20) Grade 2	0 (0)	***0.014***
Pancytopenia	0 (0)	9 (45) Grade 2–3	0 (0)	***0.001***
Leukopenia	0 (0)	9 (45) Grade 2–3	0 (0)	***0.001***
Neuropathy	0 (0)	3 (15) Grade 1–2	0 (0)	***0.042***
Pneumonitis	0 (0)	2 (10) Grade 2	0 (0)	0.126
SYMPTOM CONTROL	80% (16/20)	15% (3/20)	25% (5/20)	**0.018**

**Table 3:** Clinical results: multivariate analysis-dependent variable Cancer-Specific Survival at 1 year

ALL PATIENTS: 60				
INDEPENDENT VARIABLES:	COEFFICIENT	STD. ERROR	p-value	
(Constant)	1575		univariate	multivariate
AGE	−0,002	−0,062	0.080	0,654
SEX	0,011	0,013	0.070	0,923
PERFORMANCE STATUS	0,052	0,063	0.078	0,649
HISTOLOGY	−0,095	−0,143	0.340	0,305
STAGE	−0,049	−0,059	0.247	0,671
TREATED BULKY SITE	−0,011	−0,013	0.380	0,921
UNRESCTABLE BULKY NSCLC TUMOR DIAMETER	−0,013	-0,076	**0.047**	0,584
TREATMENT	-0,353	-0,546	**0.049**	**< 0,0001**

Abbreviations: SBRT-PATHY- SBRT PArtial Tumor irradiation of the HYpoxic segment, CHT-chemotherapy, RT- radiotherapy, CR- complete response, PR- partial response, AE- abscopal effect

Multivariate analysis (Table 3), showed that SBRT-PATHY improved cancer-specific survival (*p* < 0.001) independent of age, sex, performance status, histology, NSCLC stage, treated bulky site, and tumor diameter.

## Discussion

We present a novel concept of SBRT-PATHY to treat bulky NSCLC patients which showed improved treatment outcomes in terms of survival, tumor and symptom control, and toxicity compared to standard of care. Additionally, the duration for SBRT-PATHY (1–3 days) was shorter than the CHT arm (several months) and the palliative RT arm (2 weeks), an aspect greatly favored by both patients and hospital administrators. While therapeutic benefit of SBRT-PATHY was unequivocal, one should not forget, that it was obtained in an unfavorable patient population whose tumors were larger in size and volume than in the other two groups, and among whom 30% of patients had progressed after previous CHT.

Biologically, some of the favorable outcomes using SBRT-PATHY approach may be due to the fact that there are differences in the various states within the tumor and the surrounding peri-tumoral immune microenvironment. Our preclinical findings showed that high-single dose irradiation of exclusively hypoxic tumor cells correlated with higher probability of stronger BE/AE [30]. From this, we formulated our novel unconventional concept to treat bulky tumor partially in order to target with high fractional doses its hypoxic segment, while sparing the peritumoral immune microenvironment in order to maximize BE/AE. Indeed, a very recent retrospective analysis of the stage III NSCLC patients treated with conventional definitive RT demonstrated that higher radiation doses to the immune system were associated with increased tumor progression and death [35]. Thus, this previous work supports our efforts in trying to reduce the volume of tumor getting multiple fractions of radiation. Use of SBRT-PATHY in present study showed that lower radiation doses to the immune system were associated with improved local and distant tumor control and survival. The Italian group confirmed the efficacy of SBRT-PATHY [36].

Recent years brought renewed interest in the immuno-modulatory effects of RT, including AE [15], and the depletion of lymphocytes [16–24], both highlighting the importance of the anti-tumor immune system function. Radiation, by itself, can lead to increased antigen shedding and presentation of neo-antigens from the tumors leading to maturation and activation of antigen presenting cells (APCs), increased immuno-stimulatory cytokine production, increased CD-8 T cell and APC infiltration, and improved acquisition of immune stimulatory phenotype [37, 38]. Collectively, all of these proimmuno modulatory changes could play an important role in induction of NTE and should therefore be preserved and maintained functional. Finally, it’s getting clearer that deleterious effects of conventional whole tumor RT on loco-regional immune system could be the main reason why AE is still a rare, occasional phenomenon, and that immune-sparing effects of SBRT-PATHY were responsible for such a high rate of AE observed in our series. Based on the immunoediting hypothesis [39], it is possible that optimal radiation of only a portion of the tumor in order to avoid loco-regional immune system may be enough to initiate key alternations in tumor microenvironment to help tip the immunological shift between tumor escape towards tumor elimination. Unfortunately, no similar studies in the literature to be compared to our study results. We expect to continue with the study, and not only increase the number of patients similar to those in the presented study but also expand the indications to other tumor sites and body regions as part of an IRB approved prospective trial that is ongoing.

Our study has several shortcomings. First, it was non-randomized so it is entirely possible that various factors may have influenced treatment decision and hence, treatment outcome. Second, patient number is rather small (20 per group) which prevented us from a more detail statistical analysis. Finally, somewhat shorter follow up may have, again, limited the value of our study findings. All of these clearly call for bigger and more powerful prospective, randomized, multi-institutional studies.

## Conclusions

We provide, as far as our knowledge is concerned, the first evidence of a prospectively collected approach to treating bulky NSCLC patients using novel SBRT-PATHY. Our results, in patients that were expected to do poorly otherwise, were better than those observed in other two standard arms. We believe that there are likely immuno-modulatory effects of SBRT-PATHY that may help explain some of our results in this small phase 2 study with 60 patients. A larger prospective trial is ongoing to provide additional hope and promise for bulky, unresectable NSCLC, especially in the context of emerging immunotherapy combinations.

## Data Availability

All data generated or analysed during this study are included in this published article and its supplementary information files. Any eventual further details on the data related to this study, are available from the corresponding author on reasonable request.

## Abbreviations

AE: Abscopal effect
BE: Bystander effect
BTV: Bystander tumor volume
NTE: Non-targeted effects
PTT: Peritumoral tissue
RT-CHT: Radio-chemotherapy
SBRT-PATHY: SBRT-based partial tumor irradiation targeting hypoxic clonogenic cells
